# Physiological and morphological responses of Lead or Cadmium exposed *Chlorella sorokiniana* 211-8K (Chlorophyceae)

**DOI:** 10.1186/2193-1801-2-147

**Published:** 2013-04-08

**Authors:** Simona Carfagna, Nicola Lanza, Giovanna Salbitani, Adriana Basile, Sergio Sorbo, Vincenza Vona

**Affiliations:** 1 Dipartimento di Biologia, Università di Napoli Federico II, Via Foria 223, Naples, I-80139 Italy; 2 CISME, Università di Napoli Federico II, Via Foria 223, Naples, I-80139 Italy

**Keywords:** *Chlorella sorokiniana*, Heavy metals, OASTL activity, Photosynthesis, Respiration, TEM

## Abstract

The heavy metal pollution in soils and aquatic environments is a serious ecological problem. In the green-microalga *Chlorella sorokiniana* 211-8K (Chlorophyceae) exposed to ions of lead (Pb) and cadmium (Cd) we studied the metabolic responses to the toxicity of these two heavy metals. Our data indicate that both the pollutants alter the alga cell ultrastructure and its physiological characteristics (growth, photosynthesis, respiration, enzyme activities). The toxic effects of the two metals resulted time-dependent to the exposure. After 24 h of treatment with 250 μM Pb or Cd, photosynthesis was inhibited until to 77 and 86%, however respiration was strongly enhanced up to 300 and 350%, respectively. In the algal cells Pb or Cd exposure induced a reduction in the content of the total chlorophylls and a decrease of the soluble protein levels, significantly compromising the growth, particularly in cultures cadmium-treated. We report data on ultrastructural changes induced by the two heavy metals; they affected overall chloroplast ultrastructure of the alga. Most importantly, the O-acetyl-L-serine(thiol)lyase (OASTL) activity was appreciably increased after only 2 h of Cd exposure, indicating the existence of a link between the metal contamination and cysteine synthesis. Then, *Chlorella sorokiniana* cells seem to better tolerate high concentrations of Pb while appear to be more sensitive to Cd ions. These results provide some additional information that can lead to better understand consequences of heavy metal poisoning in microalgae.

## Introduction

Metals occur naturally, and several of them are essential components of global ecosystems. The development of human activities and industrialization has led to an increased accumulation of metals in the environment. Among the principal sources of metal pollution, there are the use of fertilizers and pesticides in agriculture (Hanikenne 2003). The contamination by heavy metals has become a serious problem because they could enter into food chains.

Microalgae, the primary producers at the base of the aquatic food chain, are the first target affected by heavy metal pollution. In microalgae trace concentrations of heavy metals are necessary as co-factor of enzymatic reactions, but high level of them could be extremely toxic (Travieso et al. 1999). Lead (Pb) and cadmium (Cd), two non-essential and toxic heavy metals for many living organisms, accumulate in algae (Debelius et al. 2009 ; Bajguz 2011). In fact, microalgae could be used to clean up contaminated water and waste streams by removing metals from soil and sediments, or solubilizing them in order to facilitate their extraction (Yoshida et al. 2006). Algae possess extracellular and intracellular mechanisms to prevent metal toxicity (Scheidegger et al. 2011). Metal uptake into algal cells is limited by lowering the metal bioavailability through excretion of non-specific ligands (Soldo et al. 2005) or by altering concentration and affinity of metal carrier proteins. Mostly important, microalgae provide important informations for predicting the environmental impact of heavy metal pollution (Akira et al. 2005).

Studies in plants (Benavides et al. 2005 ; Sharma and Dubey 2005) and algae (Szivak et al. 2009) have revealed that Pb and Cd are strongly phytotoxic, causing growth inhibition and even death (Deckert 2005); moreover, Pb induces toxic effects on protonema development and ultrastructure in the moss (Basile et al. 2008). In general, the toxic effects of metal pollutants in plant cell are related to their strong reactivity, resulting in inhibition of enzyme activity and oxidative damage. For these reasons, heavy metal ions are present in the cytoplasm mostly in a bound form (Sirko and Gotor 2007). To avoid oxidative damage, plant cells contain various antioxidant defence systems, enzymatic and non-enzymatic, designed to tightly control the concentration of ROS. Glutathione (GSH), a tripeptide containing cysteine (Cys), plays a crucial role in cellular protection (Sirko and Gotor 2007).

In the alga *Scenedesmus vacuolatus*, the exposure to Pb lowers GSH content and PC production, while Cd act as a strong inducer of PC synthesis. The same has been also observed in response to other metals and metalloids (Le Faucheur et al. 2006).

For this study we have chosen the alga *Chlorella sorokiniana*, that lives in fresh water but also in soils, (Yoshida et al. 2006 ; Chader et al. 2011), for its high capacity of adaptation during abiotic stress and its rapid growth rate (Carfagna et al. 2011a). In the present work, we focused on metabolic responses of the algal cells to Cd and Pb, two important and widespread environmental pollutants. In this study, we assessed the effect of Cd and Pb at 250 μM on growth and physiological parameters after 2 and 24 h of treatments. For the experiments we used the concentration of 250 μM, because at lower levels (50 and 100 μM) of Pb and Cd there is no significant effects (data not shown) on the algal response.

## Results

### Effect of Pb and Cd on algal growth

**Figure 1:**
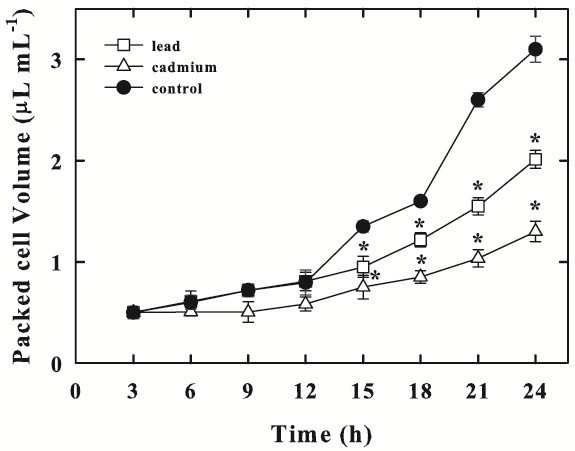
***Chlorella sorokiniana*growth, expressed as packed cell volume, in control cells (−●-), in Pb-treated (−□-) or in Cd-treated cells (−Δ-).** Data are means ± SE (n = 3). In the algal culture, Pb and Cd were supplied as 250 μM (particulars in Materials and Methods). Asterisks (*) indicate that mean values are significantly different between the treatments and the corresponding control (P < 0.05, ANOVA, Tukey multiple comparison).

The effect of exposure to either Pb or Cd on the growth of *Chlorella sorokiniana* is shown in Figure 1. The control cells exhibited a growth rate (μ), calculated in exponential growth phase, of 3.16 d^−1^. Pb or Cd exposure caused a decrease of growth rate at 2.2 and 1.65 d^−1^, respectively.

### Effect of Pb and Cd on photosynthetic rate

The photosynthetic rate in control cells of *C. sorokiniana* was initially 3.2 ± 0.26 mmol O_2_ mL^-1^ PCV h^-1^. In the Pb-treated cells, this activity was significantly reduced to 59% respect to control, reaching a value of 1.89 ± 0.25 mmol O_2_ mL^-1^ PCV h^-1^ after 2 h exposure to the metal. The photosynthesis declined progressively in time up to a value of 0.73 ± 0.05 mmol O_2_ mL^-1^ PCV h^-1^, showing an inhibition of 77%, after 24 h exposure.

**Figure 2:**
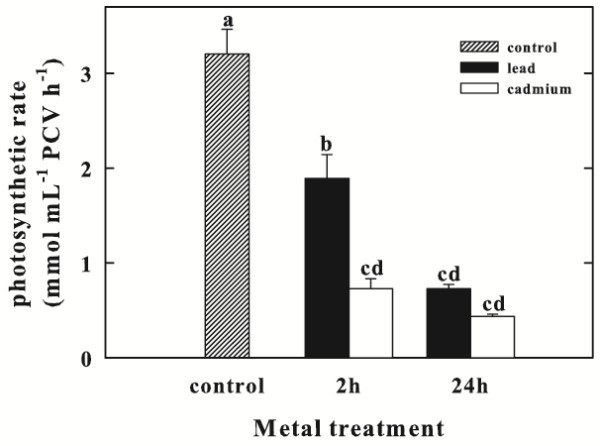
**Photosynthetic rate of*Chlorella sorokiniana*up to 24 h exposure to Pb or Cd.** Data are means ± SE (n = 3). In the algal culture, Pb and Cd were supplied as 250 μM (particulars in Materials and Methods). Columns labeled with different letters indicate statistically significant differences (P < 0.05, ANOVA, Tukey multiple comparison).

Cd supply to the cells caused a strong decrease in photosynthetic activity that decreased by 77% after 2 h of treatment reaching the value of 0.73 ± 0.1 mmol O_2_ mL^-1^ PCV h^-1^. Thereafter, the decline continued, and at 24 h, the photosynthetic rate drastically dropped to 0.44 ± 0.03 mmol O_2_ mL^-1^ PCV h^-1^, which was 14% of the control value (Figure 2).

Inhibition of photosynthesis was significantly greater in Cd-treated cells than in Pb-treated ones after 2 h.

### Effect of Pb and Cd on respiratory rate

**Figure 3:**
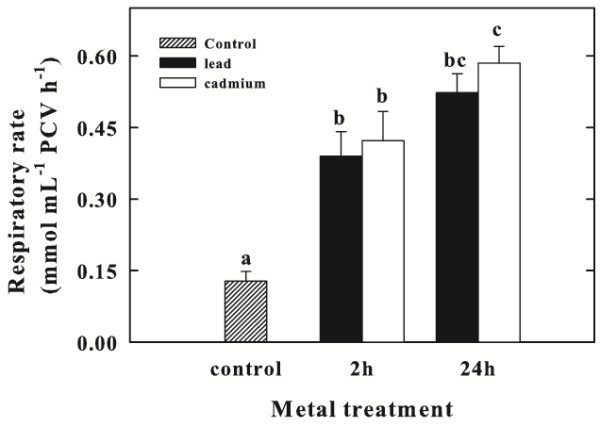
**Respiratory rate of*Chlorella sorokiniana*up to 24 h exposure to Pb or Cd.** Data are means ± SE (n = 3). In the algal culture, Pb and Cd were supplied as 250 μM (particulars in Materials and Methods). Columns labeled with different letters indicate statistically significant differences (P < 0.05, ANOVA, Tukey multiple comparison).

The respiratory oxygen consumption occurred in control cells at the rate of 0.13 ± 0.02 mmol O_2_ mL^-1^ PCV h^-1^ (Figure 3) and significantly increased upon Pb or Cd supply resulting 0.39 ± 0.05 and 0.42 ± 0.06 mmol O_2_ mL^-1^ PCV h^-1^ after 2 h, respectively. Even 24 h after the exposure to Pb or Cd, the respiratory rate was still stimulated by 4- or 4.5-fold, respectively. Again, the effect of Cd on respiration was greater than that of Pb.

### Effect of Pb and Cd on chlorophyll content

Levels of Chl were determined in Pb or Cd- treated *C. sorokiniana* cells. The effects of Pb or Cd, on Chl *a* and total Chl contents after 2 and 24 h exposures are shown in Table 1. The Chl (*a* and total) contents decreased at 2 and 24 h of treatment, compared with the control, exhibiting a similar trend in variation upon Pb and Cd exposure. The highest drop in Chl concentration occurred after 24 h of exposure to both metals.

**Table 1:** **The effect of cadmium and lead on chlorophyll*a*and total chlorophyll contents in*Chlorella sorokiniana***

		Lead		Cadmium	
**Parameter** (μg mL^-1^ PCV)	**Control**	2 h	24 h	2 h	24 h
Chl *a*	12.65 ± 0.65^a^	10.09 ± 0.35^b^	3.10 ± 0.4 ^c^	9.10 ± 0.3^bd^	2.56 ± 0.40^ce^
Total Chl	21.82 ± 0.6 ^a^	15.89 ± 1.20^b^	8.77 ± 0.35^c^	15.05 ± 0.61^bd^	7.00 ± 0.80^ce^

Data represent mean values ± SE (n = 3). Different letters in the same row mean significance of difference between the treatments (P < 0.05, ANOVA, Tukey multiple comparison).

### Effect of Pb and Cd on total protein content

**Figure 4:**
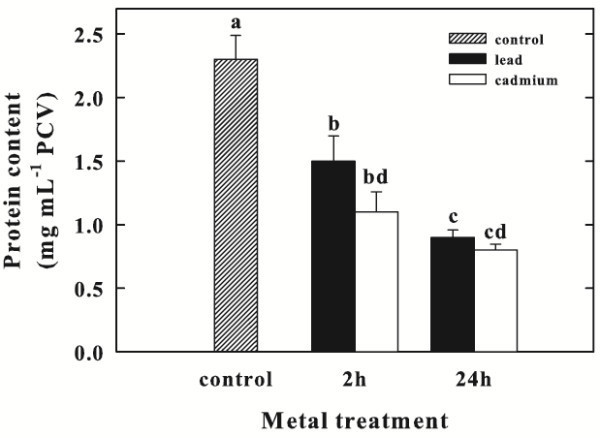
**Effect of Pb or Cd on total protein content (mg mL**^**-1**^**PCV) in*Chlorella sorokiniana*cells after 2 and 24 h.** Data are means ± SE (n = 3). In the algal culture, Pb and Cd were supplied as 250 μM (particulars in Materials and Methods). Columns labeled with different letters indicate statistically significant differences (P < 0.05, ANOVA, Tukey multiple comparison).

The soluble protein contents strongly decreased during Pb or Cd exposure (Figure 4). The protein levels in Pb-treated cells decreased, at 2 and 24 h exposure, by 35% and 61%, compared to the control. In Cd-treated cells, at 2 and 24 h exposure, the total protein levels were very low, being only 48 and 35% of the control cells, respectively.

### Effect of Pb and Cd on OASTL activity

**Figure 5:**
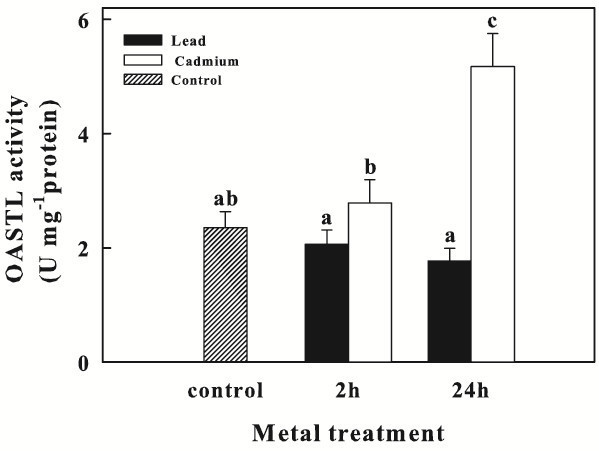
**OASTL activity (U mg**^**-1**^**protein) in*Chlorella sorokiniana*cells exposed to Pb or Cd for 2 and 24 h.** Data are means ± SE (n = 6). In the algal culture, Pb and Cd were supplied as 250 μM (particulars in Materials and Methods). Columns labeled with different letters indicate statistically significant differences (P < 0.05, ANOVA, Tukey multiple comparison).

The OASTL activity was determined in *C. sorokiniana* control cells and in Pb or Cd treated cells (Figure 5). The enzyme activity seems to be not influenced by lead treatment for 2 and 24 h; on the other hand, Cd exposure caused an significant increase in OASTL specific activity from 2.35 ± 0.28 U mg^-1^ protein to 2.8 ± 0.41 U mg^-1^ protein after 2 h and up to 5.2 ± 0.57 U mg^-1^protein after 24 h.

### TEM

**Figure 6:**
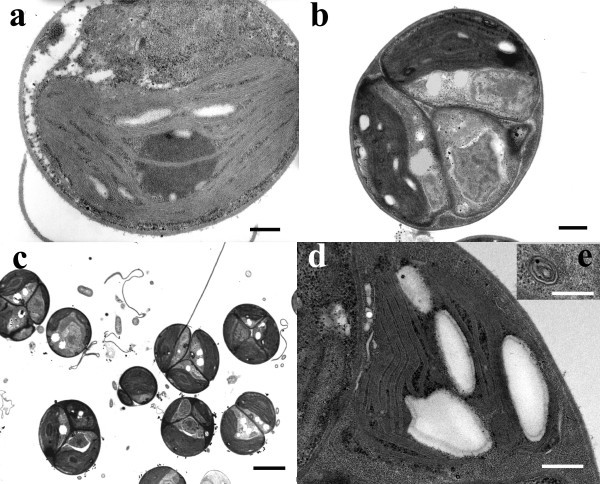
***Chlorella sorokiniana*TEM micrographs: control (a), Pb-treated (b, c) and Cd-treated (d, e) culture.a**. A cell with a cup-shaped chloroplast containing thylakoids, a central pyrenoid, and electron clear starch grains. **b**. A colony of algal cells with misshaped chloroplasts, a nucleus, and cytoplasm lipid droplets. **c**. The typical appearance of a Pb-treated culture with cells organized as colonies. **d**. A cell with a shape-altered chloroplast still containing thylakoids and starch grains. **e**. A multivesicular body. Scale bars: 2 μ (**c**); 500 nm (**b**, **d**); 300 nm (**a**, **e**).

Control cells showed a typical ultrastructure, with a cup-shaped chloroplast containing thylakoids arranged along the main axis of the organelle, a central pyrenoid, and electron clear starch grains (Figure 6a). Nucleus and cytoplasm showed a typical organization. No vesiculations were visible in the cytoplasm, which contained electrondense masses of reserve materials.

After a 24 h Pb-treatment, mostly of the cells appeared as colonies of four- and more cells with a common cell wall (Figures 6b and c). The ultrastructural organization showed deformed chloroplasts, with an amoeboid shape, containing thylakoids distributed along the main axis of the organelle, and a central pyrenoid. Numerous lipid droplets and cytoplasm vesicles were visible in the cytoplasm. Nucleus didn’t show ultrastructural alterations.

After a 24 h Cd-treatment, the cells showed altered chloroplast with an amoeboid shape and untidily arranged thylakoids (Figures 6d and e). Chloroplasts still contained starch grains. Pyrenoid was lacking or small. Nucleus and cytoplasm didn’t show differences in comparison to control, except for the occurrence of cytoplasm vesicles and multivesicular bodies (Figure 6e).

## Discussion

Despite an increasing literature on biochemical events in response to heavy metals stress in higher plants, little is known about unicellular algae. Microalgae play an important role in the equilibrium of aquatic ecosystems and represent highly suitable biological indicators of environmental changes.

We utilized a strain of *Chlorella sorokiniana*, an unicellular chlorophyte, as a model system for physiological, biochemical and morphological studies on heavy metal stress response. The algal cells were exposed for a short and a long period (2 and 24 h) to heavy metal such as Cd and Pb (250 μM).

Cd and Pb inhibited the specific growth rate. In Pb-treated samples we observed most of the algal cells grew as colonies and contained plenty of cytoplasm lipid droplets. Probably, *Chlorella* cells, after first mitosis, continue to divide, forming colonies of four- and more cells with a common cell wall, and only some algae live independently.

The occurrence of adipocyte forms deriving from starch granules and lipids, even after a short time treatment, is regarded as stress symptoms (Lebsky 2004).

Concentric multilamellar/multivesicular bodies were well distinct in *Chlorella* cells Cd-polluted. These organelles were regarded as accumulation of membranes correlated with incomplete digestion of endocytosed material: double-membrane-bound vesicles arising from endoplasmic reticulum tubes enclose cytoplasm or organelles and coalesce to seal the region in a double-membrane-bound compartment.

We suggest that the repeated phenomena of encircling and sealing cytoplasmic regions by endoplasmic reticulum tubes might generate the concentric multilamellar bodies in our cadmium exposed samples.

Thompson and Vierstra (2005) reported the autophagic activity of cell vacuoles to destroy the cytoplasm and nucleus. Autophagocytosis phenomena have been reported also for unicellular algae (Reunova et al. 2007).

We suggest that the cytoplasm vesicles, and concentric multilamellar/multivesicular bodies were induced by heavy metal treatment and they might be involved in the transport of metal ions to vacuoles or vesicles and/or in the elaboration of massive newly formed or damaged material.

In *Chlorella*, Cd had a much higher effect than Pb on physiological functions such as photosynthesis and respiration. Particularly, photosynthetic activity resulted strongly compromised by Cd treatment. According to Neelam and Rai (2003), a possible explanation for Cd effect on photosynthesis could be the inhibition of the PS II as a result of damage of thylakoid membranes and reaction centers. In fact, *Chlorella* cells showed an ultrastructural alteration in the shape and organization of thylakoids that confirm a damage of the photosynthetic apparatus. Chloroplasts and their arrangement represent a common target of toxic substances in algae and higher plants (Carginale et al. 2004 ; Nacorda et al. 2007 ; Basile et al. 2008). The altered chloroplast shape could be a consequence of a perturbation of cation exchange induced by heavy metal-treatment (Basile et al. 2008), while the structural alterations of the thylakoid system could depend on the ability of heavy metals to bind to proteins and to interfere with their normal functions, also inducing oxidative damage (Heumann 1987). Moreover, the chloroplast alterations in leaves of tomato exposed to heavy metals were related to an increase in the production of ROS (Gratao et al. 2009). In our experiments, associated to ultrastructural alterations of plastids, we noted a significant reduction of the photosynthetic rate related to a decrease of total Chl and Chl *a* contents that both pollutants Pb and Cd caused. The decreases of Chl *a* and total Chl indicated a decline in the antenna size of the photosynthetic reaction center complexes. Qiu et al. (2006) showed that in *Chlorococcum* sp. AZHB the decrease of Chl *a* correlated to the increasing concentrations of Cu or Cd treatment. The decrease of chlorophyll, accompanied by the degradation of the chloroplast structure, in *Chlorella* heavy metal polluted indicates that the photosynthetic apparatus in these cells could be disrupted.

Therefore, the decrease of growth occurring in the algae Cd or Pb treated could be ascribed to the reduction of the photosynthetic activity.

A decrease of soluble protein content was detected in *C. sorokiniana* cells Pb- or Cd-treated for 24 h by 61 and 65% of the control, respectively. Probably, chlorophylls and proteins, and even the chloroplast proteins, represented an emergency source of nitrogen and sulfur to ensure cell growth. Moreover, the reduction of protein content might also be attributed to the shortage of carbon skeleton resulting from low photosynthetic rate.

The respiratory rate of the alga, under Pb or Cd exposure significantly increased. Little is known in literature about heavy metal-induced cellular respiration. Desouky (2011) reported that the respiration of pollutant Chlorella vulgaris cultures was considerably increased by heavy metal exposure: but these studies were conducted in algae treated with high (6 and 8 ppm) and low (2 and 4 ppm) concentrations of CoCl_2_ or NiCl_2_.

The remarkable enhancement of respiratory oxygen consumption in *C. sorokiniana* cells exposed to Pb or Cd could be an adaptive developed strategy to compensate the energy depletion caused by a low photosynthetic activity. *C. sorokiniana* cells seemed to use respiration to provide more energy to sustain the metabolism as previously reported (Vona et al. 1999).

Most studies regarding oxidative stress responses indicate Cys and GSH as the main antioxidants in the plant cell apart from ascorbate (Foyer and Noctor 2009).

In this study, we reported that in *C. sorokiniana* cells Cd-treated, OASTL activity enhanced up to 2.2-fold after 24 h, respect to control cells. However, the activity of this enzyme seems to be not influenced by Pb treatment. The different results on OASTL activity between Cd and Pb treatment could depend on the toxicity level of the single metal. Like in the epiphytic moss *Scorpiurum circinatum* (Carfagna et al. 2011b), the positive relationship concerning OASTL and metal exposure may suggest that in *Chlorella* cells exists a link between heavy metal tolerance and Cys synthesis. Increased Cys synthesis associated with heavy metals appears to be a necessary response for biosynthesis of GSH and of the other ligands involved in the heavy metal binding.

## Conclusion

The present study provided data on the toxicity of Pb and Cd on the cell ultrastructure and physiology of the green-microalga *Chlorella sorokiniana*. Our data suggest that the toxic effects of the two heavy metals resulted time-dependent to the exposure. Furthermore, the exposure of the algae to Cd or Pb compromises the cell growth rate.

These two heavy metals provoke a strong inhibition of photosynthesis but a significant enhancement of respiratory rate, as well as a reduction in the content of the total chlorophylls and of the soluble protein levels. The OASTL activity increase in Cd-treated cells suggests the existence of a link between the metal contamination and cysteine synthesis. *Chlorella sorokiniana* cells seem to better tolerate high concentrations of Pb while appear to be more sensitive to Cd ions. These results provide some additional information about the effects of the heavy metals in microalgae.

## Materials and methods

### Strain and culture conditions

*Chlorella sorokiniana* Shihira & Krauss, strain 211/8K (CCAP of Cambridge University), was grown in batch culture at 35°C, continuously illuminated (Philips TLD 30 W/55 fluorescent lamps, 250 μmol photons m^-2^ s^-1^), and flushed with air containing 5% CO_2_ at a flow rate of about 80–100 l h^-1^. The composition of the basal medium (pH 6.5) and the growth procedure were previously reported (Vona et al. 2004). The nitrogen source was supplied as 10 mM KNO_3_. Under these conditions, the growth rate constant (μ), measured on the basis of variations in packed cell volume **(**PCV), was 3 d^-1^. For further experiments, cells were collected during exponential growth phase.

### Metal treatments

Experiments were performed using the following analytical grade salts: cadmium chloride hydrate (CdCl_2_ · H_2_O) and lead acetate [Pb(CH_3_COO)_2_]. Cell suspensions of *C. sorokiniana* were exposed to 250 μM of the two tested metal salts. Samples of the cell suspension, harvested after 2 h and 24 h from the beginning of the metal exposure, were used for different determinations as indicated in the text. Algae cultured in the nutrient medium without heavy metals were used as controls.

### Growth and Packed cell volume (PCV) determination

Algae growth was measured as PCV by centrifuging a known aliquot of cell suspension in a haematocrit tube at 4000 × g for 5 min (Centrifuge Thermo CL10).

The specific growth rate (μ) was calculated for the exponential growth phase as ln[(PCV_t_/PCV_0_)/Δt], where PCV_0_ is the initial PCV, PCV_t_ is the final PCV, and Δt is the time interval between biomass measurements.

### Photosynthetic and respiratory rates

Photosynthesis and respiration were measured as O_2_ exchange in a glass biochemical oxygen demand (BOD) water-jacketed bottle equipped with an oxygen electrode (Orion 97–08), connected to an EA 920 ion analyser.

To measure photosynthesis, cells were collected during exponential growth by centrifugation and re-suspended to a final concentration of about 0.5 μl PCV mL^-1^ in a fresh basal medium supplemented with 10 mM NaHCO_3_ to prevent carbon limitation, then transferred to the oxygen electrode chamber, illuminated with incandescent light (Philips Comptalux 300 W 13736 E/44, the Netherlands), 1000 μmol photons m^-2^ s^-1^, sufficient to achieve full photosynthesis in the alga. Photon flux density inside the culture was measured using a quantum meter with a separate sensor (model Q MSS-SUN) (Apogee Instruments, Logan, UT, USA). The medium in the chamber was magnetically stirred.

To measure respiration, the BOD bottle was obscured by covering it with aluminum foil. Photosynthetic oxygen evolution and respiratory oxygen consumption were expressed as mmol O_2_ mL^-1^ PCV h^-1^. The rate of gross photosynthesis was derived from the algebraic sum of the rate of the net photosynthetic O_2_ evolution and the rate of respiration.

### Chlorophyll content

The chlorophyll (Chl) content (*a* and total) was estimated spectrophotometrically after extraction with N,N-dimethylformamide according to Inskeep and Bloom (1985).

### Assay of OASTL activity

*C. sorokiniana* cells (500 mL of suspension), were harvested by low-speed centrifugation (4000 × g for 5 min), re-suspended in cold extraction buffer: 50 mM potassium phosphate buffer (pH 7.5), 1 mM dithiothreitol, 10 μM pyridoxal 5’-phosphate, and then broken by passing twice through a French pressure cell (11,000 psi). The homogenate was centrifuged (Sorvall RC5C – Sorvall Rotor SS34) at 16,000 × g for 20 min at 4°C, and the clear supernatant was used as crude extract. Enzymatic OASTL activity was determined colorimetrically, measuring the cysteine formed, as described in Carfagna et al. (2011a). One unit (U) of OASTL activity corresponds to the formation of 1 μmol of Cys min^-1^. The OASTL activity was related to the soluble protein content of the samples.

### Soluble protein determination

The protein concentration was determined according to Bradford (1976), using bovine serum albumin as standard.

### Ultrastructural observations

After collection, the algae samples were prepared for Transmission Electron Microscopy (TEM) observations as detailed in Basile et al. (1994). Briefly, after fixation with 3% glutaraldehyde for 2 h at room temperature, samples were post-fixed in 1% OsO_4_ at 4°C, before being dehydrated with ethanol. After dehydration, samples were embedded in Spurr’s epoxy resin. Ultrathin sections (60 nm) were cut with a diamond knife on a Supernova microtome and sequentially stained at room temperature with uranyl acetate (3%) for 12 min and Reynold’s lead citrate (2%) for 8 min. A FEI EM 208S TEM, with an accelerating voltage of 80 kV, was employed for observations.

### Statistical analyses

The data were analyzed by two-way analyses of variance (ANOVA) followed by a Tukey test to determine whether significant differences occurred between treatments. Differences were considered significant at P < 0.05. All analyses were carried out using SigmaPlot (version 11.0).

### Reagents

All reagents used are of analytical grade and purchased from the Sigma-Aldrich (Milan, Italy). The solutions were prepared with ultrapure water (Millipore). All reagents and chemicals were used without any further purification.
